# Transcriptome and Metabolome Reveal Distinct Sugar Accumulation Pattern between PCNA and PCA Mature Persimmon Fruit

**DOI:** 10.3390/ijms24108599

**Published:** 2023-05-11

**Authors:** Weijuan Han, Yiru Wang, Huawei Li, Songfeng Diao, Yujing Suo, Taishan Li, Peng Sun, Fangdong Li, Jianmin Fu

**Affiliations:** Research Institute of Non-Timber Forestry, Chinese Academy of Forestry, Zhengzhou 450003, China; hanweijuan2013@163.com (W.H.); wangyiru199702@163.com (Y.W.); lihuaweicaf@163.com (H.L.); dsf@caf.ac.cn (S.D.); suoyujing1988@126.com (Y.S.); litaishan910706@gmail.com (T.L.); sunpeng1017@126.com (P.S.)

**Keywords:** persimmon, soluble sugar, starch and sucrose metabolism, transcriptome, metabolome

## Abstract

Persimmon (*Diospyros kaki*) fruit have significant variation between pollination-constant non-astringent (PCNA) and pollination-constant astringent (PCA) persimmons. The astringency type affects not only the soluble tannin concentration but also the accumulation of individual sugars. Thus, we comprehensively investigate the gene expression and metabolite profiles of individual sugars to resolve the formation of flavor differences in PCNA and PCA persimmon fruit. The results showed that soluble sugar, starch content, sucrose synthase, and sucrose invertase were significantly different between PCNA and PCA persimmon fruit. The sucrose and starch metabolism pathway was considerably enriched, and six sugar metabolites involving this pathway were significantly differentially accumulated. In addition, the expression patterns of diferentially expressed genes (such as *bglX*, *eglC*, *Cel*, *TPS*, *SUS*, and *TREH* genes) were significantly correlated with the content of deferentially accumulated metabolites (such as starch, sucrose, and trehalose) in the sucrose and starch metabolism pathway. These results indicated that the sucrose and starch metabolism pathway maintained a central position of sugar metabolism between PCNA and PCA persimmon fruit. Our results provide a theoretical basis for exploring functional genes related to sugar metabolism and provide useful resources for future studies on the flavor differences between PCNA and PCA persimmon fruit.

## 1. Introduction

The persimmon (*Diospyros kaki*), a plant of the family Ebenaceae, has a long history of cultivation [1]. The persimmon, as a major fruit variety, has a high commercial value in Asian countries. In China, the production of persimmon fruit was approximately 3,429,438 tonnes in 2021, which accounted for 79.16% of the total production around the world. The genetic characteristics of fruit de-astringency allow for the categorization of persimmon into PCNA (pollination-constant non-astringent), PCA (pollination-constant astringent), pollination-variant non-astringent (PVNA), and pollination-variant astringent (PVA) types [2]. More than 950 cultivars are now recognized in China, practically all of which belong to the PCA type; no PVA and PVNA varieties have been identified [3].

Soluble sugars mainly comprise fructose, sucrose, and glucose in fruit [4]. Fruit can be categorized into the hexose-accumulating type, sucrose-accumulating type, and intermediate type according to the sugar composition in mature fruit [5,6]. Fruit sugar metabolic processes are complex [7]; according to the different substrates, sugar metabolic processes can be divided into sorbitol, sucrose, hexose-type sugar, and starch metabolism [8]. Sucrose metabolism is the basis of the fruit sugar metabolism; it directly affects fruit sugar accumulation [4] and is vital in forming fruit quality [9]. In the fruit of Rosaceae trees, sorbitol is important in translocating photosynthate [10]. Sucrose and sorbitol can be converted to glucose, fructose, and starch after entering the fruit, catalyzed by a range of enzymes implicated within sugar metabolic processes [4,11]. An essential enzyme in sucrose production, sucrose phosphate synthase (SPS), transforms fructose-6-phosphate into sucrose phosphate [12]. Sucrose synthase (SS) is a bifunctional enzyme that synthesizes/hydrolyzes sucrose [13]. The enzymes are classified according to their optimum pH into neutral invertase (NI) and acid invertase (AI) [14], which catalyze the sucrose decomposition into fructose and glucose [15].

Persimmon fruit have significant variation between PCNA and PCA types [16,17]. The astringency type influences flavonoid metabolite content, soluble tannin concentration, individual sugar accumulation, and antioxidant capacity [17,18]. Thus, the differences in sugar metabolism are crucial for resolving flavor formation between PCNA and PCA persimmon fruit. This investigation probed sugar accumulation variation across PCNA/PCA fully matured fruit by analyzing sugar content and enzyme activities between mature PCNA varieties (‘Yohou’ and ‘Jiro’) and PCA fruit varieties (‘Zhongshi5’). We also examined the gene expression patterns and the metabolites’ accumulation patterns in the sucrose and starch metabolism pathway through transcriptomic and metabolomic analyses. The findings of this study may provide basic information on the sugar accumulation of persimmons and facilitate a flavor formation analysis between PCNA and PCA persimmons.

## 2. Results

### 2.1. Soluble Sugar Content, Starch Content, and Sucrose Synthase and Invertase Activity

**Figure 1:**
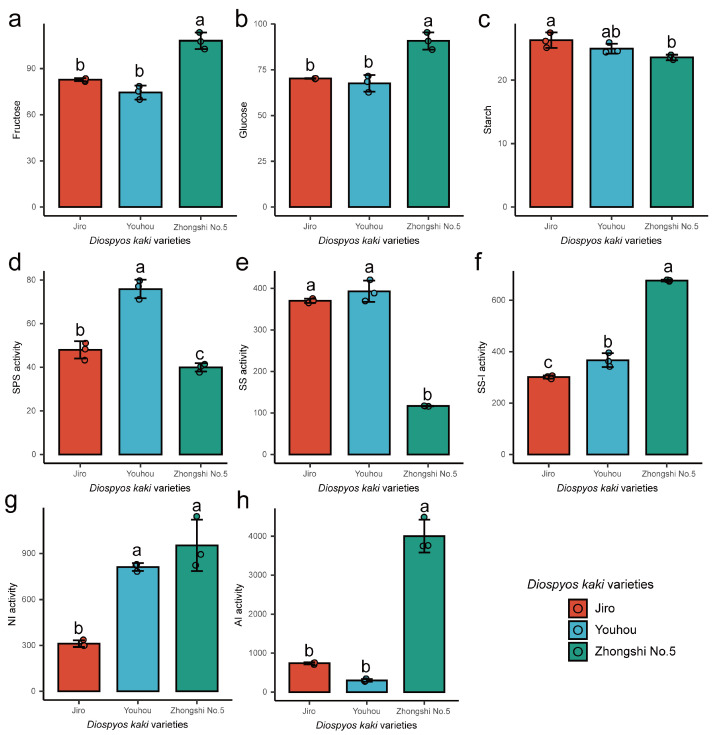
Soluble sugar content, starch content, and key enzymes within sugar metabolism in mature PCNA (‘Jiro’ and ‘Youhou’) and PCA (‘Zhongshi No.5′) persimmon fruit. (**a**–**h**) Fructose, glucose, starch, SPS, SS, SS-I, AI, and NI, accordingly. Significant variations (*p* < 0.05) are represented as lowercase letters. All error bars illustrate SD for the mean (*n* = 3).

For evaluating the differences in sugar accumulation between PCNA and PCA persimmons, soluble sugar fructose and glucose concentration, starch concentration, and sucrose synthase (SPS and SS) and invertase (SS-I, AI, and NI) activities were compared in mature PCNA (varieties ‘Jiro’ and ‘Youhou’) and PCA (variety ‘Zhongshi No.5’) persimmon fruit (Figure 1). The fructose, glucose, SS-I, and AI activity levels in the PCNA persimmon fruit were markedly lower than those in the PCA persimmon fruit. The SS activity was 3.2-fold and ~3.4-fold higher within PCNA persimmon fruit compared to PCA persimmon fruit. The starch content was slightly higher within PCNA persimmon fruit compared to PCA fruit. Overall, the soluble sugar, starch content, and sucrose synthase and invertase of PCNA and PCA persimmon fruit were significantly different; thus, soluble sugar accumulation may be one of the important reasons for the flavor differences between PCNA and PCA persimmon fruit.

### 2.2. RNA-Seq of PCNA and PCA Persimmon Mature Fruit

**Figure 2:**
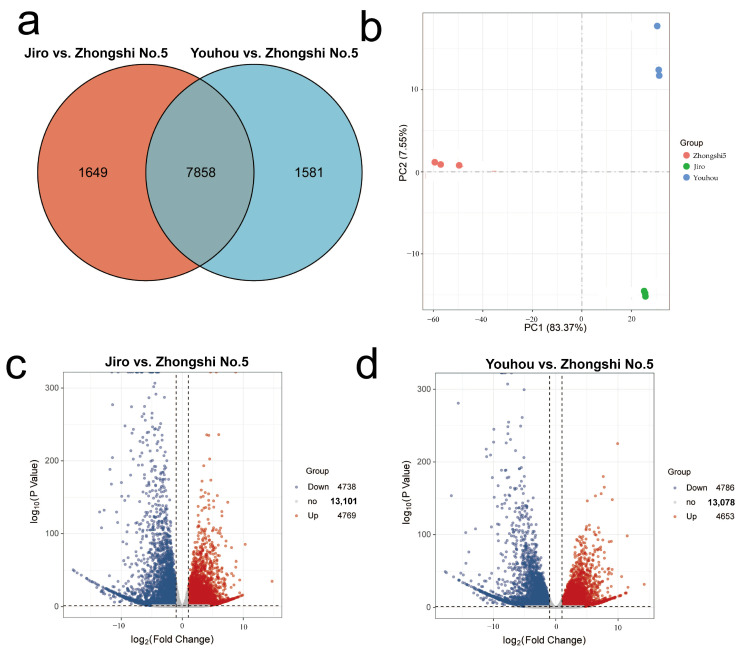
PCNA and PCA persimmon mature fruit transcriptome. (**a**) Venn diagram of unique/common DEGs between Jiro vs. Zhongshi No.5 and Youhou vs. Zhongshi No.5. (**b**) Principal component analysis (PCA) of Jiro vs. Zhongshi No.5 and Youhou vs. Zhongshi No.5. The samples from the same varieties were grouped. (**c**) The volcano plot shows the number of DEGs in Jiro vs. Zhongshi No.5 (|log2-fold change| > 1 and padj < 0.05). (**d**) DEG number in Youhou vs. Zhongshi No.5 was shown by the volcano plot (|log2-fold change| > 1 and padj < 0.05).

‘Jiro’, ‘Youhou’, and ‘Zhongshi No.5’ matured persimmon fruit transcriptome sequencing produced 62.88 GB of raw data. Each sample had 6.99 GB of high-quality data with a Q30 score at 92.52% after the low-quality reads were removed. A total of 4417 new unique transcripts were also discovered, and 86.05% of reads could be mapped onto the reference *D. kaki* genome. This demonstrates that the sequencing data’s precision and quality were good enough for further investigation. The samples were segregated into three distinct groups using PCA depending upon FPKM values, with each sample creating a separate group with its replicates. This revealed strong correlations within sample replicates and variations among different samples (Figure 2a).

To examine expression-profile differences linked to sugar accumulation between PCNA and PCA persimmons, the genes in nine libraries (Jiro vs. Zhongshi No.5 and Youhou vs. Zhongshi No.5) were compared. A total of 11,088 genes were substantially different in pairwise comparisons, with 9507 DEGs in Jiro vs. Zhongshi No.5 and 9439 DEGs in Youhou vs. Zhongshi No.5. A total of 7858 DEGs were differently expressed in both comparisons, according to the Venn diagram (Figure 2b–d). By comparing the RT-qPCR assessment of nine sugar-accumulation-associated DEGs together with transcriptomic FPKM datasets, a gene expression pattern consistent with the transcriptomic data was shown (Figure S1).

### 2.3. GO and KEGG Enrichment Analysis for DEGs

**Figure 3:**
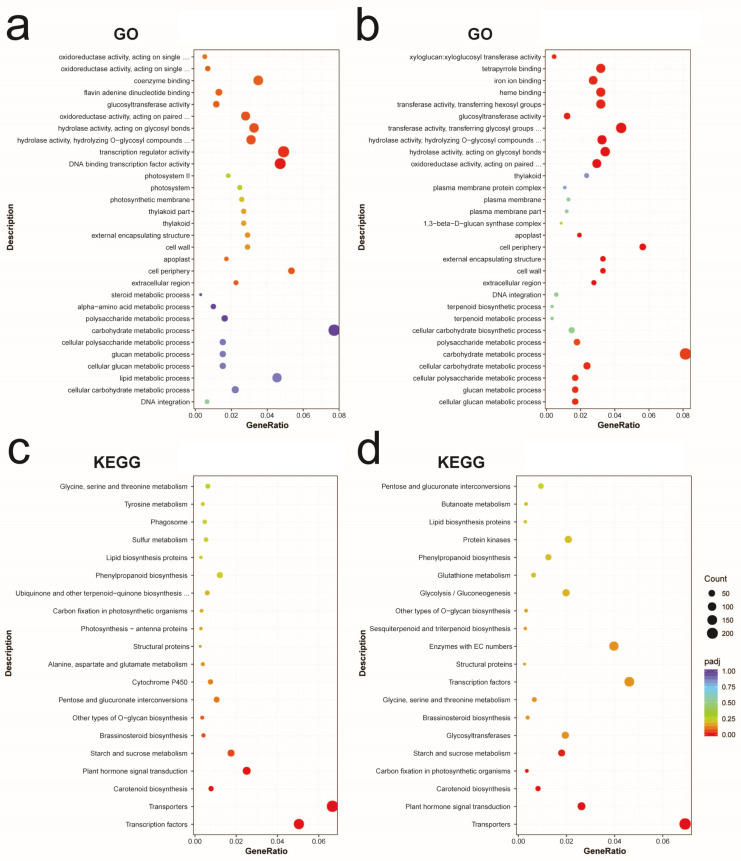
DEGs in PCNA and PCA. DEG enrichment GO enrichment scatter plot for (**a**) Jiro vs. Zhongshi No.5 and (**b**) Youhou vs. Zhongshi No.5. DEG enrichment KEGG scatter plot for (**c**) Jiro vs. Zhongshi No.5 and (**d**) Youhou vs. Zhongshi No.5.

Further analysis of DEGs in the comparison groups Jiro vs. Zhongshi No.5 and Youhou vs. Zhongshi No.5 was performed using the GO and KEGG databases; padj < 0.05 represented a significant difference. The DEGs in the comparison of Jiro vs. Zhongshi No.5 were mainly enriched in two GO terms: DNA-binding transcription regulator activity (GO:0140110) and transcription factor activity (GO:0003700). The DEGs in the comparison of Youhou vs. Zhongshi No.5 were mainly enriched in several different terms, such as the glucan metabolic process (GO:0044042), cellular glucan metabolic process (GO:0006073), cellular polysaccharide metabolic process (GO:0044264), cellular carbohydrate metabolic process (GO:0044262), and carbohydrate metabolic process (GO:0005975) (Figure 3a,b).

Furthermore, KEGG enrichment analysis showed that the DEGs in Jiro vs. Zhongshi No.5 were significantly enriched in transcription factors, transporters, plant hormone signal transduction, carotenoid biosynthesis, and sucrose and starch metabolism. The DEGs in the comparison between Youhou and Zhongshi No.5 were significantly enriched in transporters, plant hormone signal transduction, carotenoid biosynthesis, carbon fixation in photosynthetic organisms, starch and sucrose metabolism, and biological processes (Figure 3c,d). Though the enrichment analysis, sucrose and starch metabolic processes were highly enriched between the two combinations. To understand the differences in sugar accumulation between PCNA and PCA persimmon fruit, the starch and sucrose metabolism were considered for downstream analysis (Figure 3).

### 2.4. Metabolic Features of Starch and Sucrose Metabolism

**Figure 4:**
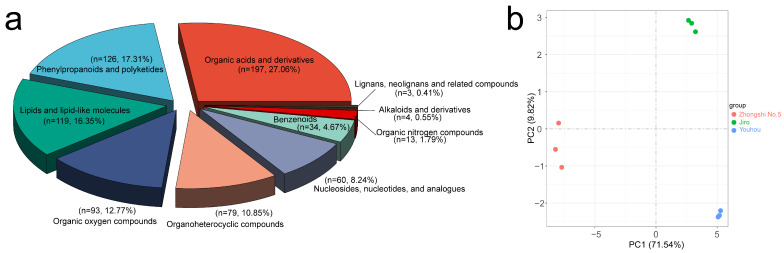
Analysis of metabolites: (**a**) statistics on the types of metabolites; (**b**) PCA of Jiro vs. Zhongshi No.5 and Youhou vs. Zhongshi No.5. The samples from the same varieties were grouped.

A total of 728 compounds were identified throughout quasi-targeted metabolome analysis, allowing us to characterize metabolic changes in mature PCNA (‘Jiro’ and ‘Youhou’) and PCA (‘Zhongshi No.5’) persimmon fruit (Table S2). Organic acids and derivatives (197); phenylpropanoids and polyketides (126); lipids and lipid-like molecules (119); organic oxygen compounds (93); organic heterocyclic compounds (79); nucleosides, nucleotides, and analogs (60); benzenoids (34); and organic nitrogen compounds (13) were all included in the eight major categories of metabolites (Figure 4a). There were apparent similarities within sample replicates and differences between the samples, as shown by the PCA analysis results based on intensity values for metabolites, which clustered samples into three groups (Figure 4b).

For starch and sucrose metabolism, 11 metabolites were identified, including UDP-D-glucose, GDP-α-D-glucose, sucrose, isomaltose, maltose, D-glucose 1-phosphate, α,α-trehalose, trehalose 6-phosphate, D-glucose 6-phosphate, D-(+)-Cellobiose, D-glucopyranose, and D-glucose (Table S3). PCNA (‘Jiro’ and ‘Youhou’) persimmon fruit differed from PCA (‘Zhongshi No.5’) persimmon fruit in sucrose, maltose, D-(+)-Cellobiose, D-glucose 1-phosphate, α,α-trehalose, and trehalose 6-phosphate content. The contents of sucrose, maltose, and trehalose 6-phosphate in PCA persimmon fruit were markedly higher than compared to the two PCNA varieties of fruit, while D-glucose 1-phosphate contents were markedly reduced compared to PCNA fruit. Most of these differentially expressed sucrose metabolites were related to trehalose synthesis, a module of the starch and sucrose metabolism pathway, indicating that the trehalose synthesis pathway might influence the sugar accumulation between PCNA and PCA persimmon fruit.

### 2.5. Expression of Genes Implicated within Sucrose and Starch Metabolic Pathway

**Figure 5:**
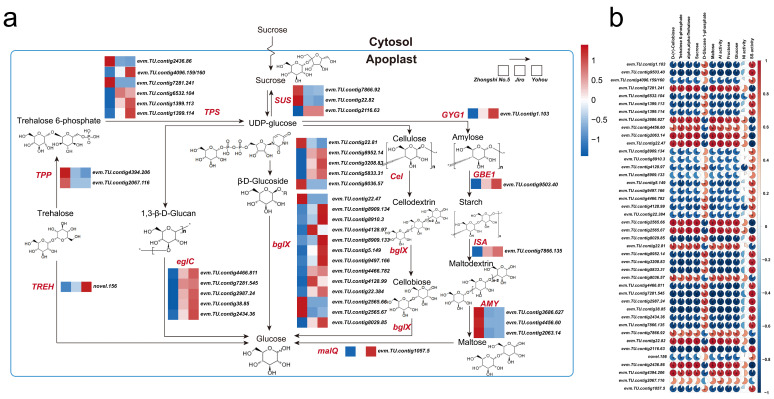
DEG analysis of sucrose and starch metabolic processes between PCNA and PCA: (**a**) diagram of DEGs implicated within sucrose and starch metabolic processes between PCNA and PCA; (**b**) correlation analysis between sucrose and starch metabolic pathway DEGs and metabolite content in PCNA and PCA persimmon fruit.

Fifty-eight DEGs related to the starch and sucrose metabolic pathway that encode 11 key enzymes were identified (Figure 5). These enzymes include granule-bound starch synthase (EC:2.4.1.242), 1,4-alpha-glucan branching enzyme (EC:2.4.1.18), trehalose 6-phosphate synthase (2.4.1.15), Alpha-amylase and beta amylase (EC:3.2.1.1; 3.2.1.2), beta-glucosidase (EC:3.2.1.21), glucan endo-1,3-beta-Dglucosidase (EC:3.2.1.39), sucrose synthetase (EC 2.4.1.13), alpha,alpha-trehalase (EC:3.2.1.28), trehalose 6-phosphate phosphatase (EC:3.1.3.12), and 4-alpha-glucanotransferase (EC:2.4.1.25). Out of 58 genes, 42 were differentially expressed in both comparison groups, Jiro vs. Zhongshi No.5 and Youhou vs. Zhongshi No.5. Ten bglX, five eglC, four Cel, four TPS, one GBE1, one GYG1, one malQ, one SUS, and one TREH genes were upregulated in Jiro and Youhou in comparison with Zhongshi No.5, though the other genes had reduced expression levels. Differing transcripts from identical genes were dysregulated, suggesting that intermediate products may be being converted between each other (Figure 5a). Sugar accumulation may be facilitated by gene expression implicated within the sucrose and starch metabolic pathway since there was a robust association between their expression and metabolite levels in PCNA and PCA persimmon fruit (Figure 5b).

Combined KEGG analysis and gene expression patterns showed that widespread shifts within sucrose and starch metabolic processes mainly concentrated on converting sucrose into glucose. These changes indicate that converting sucrose into glucose might influence sugar accumulation between PCNA and PCA persimmon fruit.

## 3. Discussion

Quality improvement and efficiency are currently important issues for the persimmon industry, and flavor is the most important component of persimmon fruit quality, which has an important impact on both the fresh and processed quality of persimmon fruit. The fruit flavor quality varies greatly among different types and varieties, and there are few germplasm resources with excellent flavor. In this study, we analyzed the differences in the accumulation of various sugars and the differences in gene expression and enzyme activities related to sugar metabolism in different PCNA and PCA types of persimmon varieties, to further provide basic information on the sugar accumulation between PCA and PCNA persimmon fruit and facilitate a flavor formation analysis between PCNA and PCA persimmons.

Glucose and fructose are generally considered the main sugars, while sucrose is present as a minor component in mature persimmon fruits [19,20]. The HPLC method is less sensitive and has a higher detection limit, while the ultra-performance liquid chromatography coupled with triple quadrupole mass spectrometer has a lower detection limit and can detect compounds generally greater than 10 ng/mL, allowing a broad-spectrum determination of compounds in plant samples. Thus, the fructose and glucose contents were determined by HPLC, and the sucrose was determined by LC/MS-MS due to its low content in this study. The fructose and glucose levels of PCNA cultivars were lower than those of non-PCNA cultivars in tree maturity, which agrees with the work by Yildiz et al. [17]. Changes in the content of soluble sugars in fruits were closely related to their metabolic enzyme activities. By measuring the content of sugar fractions and enzyme activities related to sucrose metabolism during fruit ripening in different astringent-type persimmon varieties, it was shown that SS, SS-I, and AI activities differed significantly in PCNA and PCA persimmons. Sucrose metabolism in red Fuji apple fruit was mainly regulated by AI and SS enzyme activities [21]. Studies on ‘Malaysia 1’ pineapple honey found that sucrose accumulation was significantly negatively correlated with AI activity and significantly positively correlated with SS and SPS activities [22], in agreement with studies on grapes [23]. Bubba et al. [24] showed that sucrose content peaked at the beginning of October and then gradually decreased, while glucose and fructose content showed an increasing trend throughout development. The above studies suggest that sucrose synthesis decreases and catabolism increases in PCNA and PCA persimmons before ripening, which results in an increase in soluble sugar content in ripe persimmon fruit. In addition, the difference in resource enzyme activity changes between PCNA and PCA persimmons eventually led to a lower soluble sugar content in PCNA persimmon fruit than in PCA persimmons.

The post-harvest conversion of starch to sugar improves fruit quality and flavor [25,26,27,28]. KEGG analysis showed that the two comparative combinations of sweet and astringent persimmons were significantly enriched in starch and sucrose metabolism. Therefore, analysis for differences in metabolites on the starch and sucrose pathways revealed that PCNA (‘Jiro’ and ‘Youhou’) persimmon fruits differed from PCA (‘Zhongshi No.5′) persimmon fruit in trehalose and tre6p content. The trehalose metabolic pathway is involved in processes such as cell wall cellulose synthesis, energy release from respiration, and the carbon skeleton composition of amino acids and fatty acids, and it is central to cellular metabolism [29]. Tre6P is a precursor of trehalose metabolism, employing UDP-glucose and glucose-6-phosphate as substrates, which *TPS* catalyzes to produce Tre6P; subsequently, Tre6P is dephosphorylated by *TPP* to produce trehalose [30]. Tre6P regulates various vital activities of tissues by effectively regulating the content of UDPG and G6P, reflects a vital signal for controlling plant growth and development [31], and is implicated within metabolic/genomic expression regulation within plants [32]. Tre6P acts as a signaling metabolite for sucrose; its content exists in proportion to sucrose content and affects starch synthesis [33,34]. Tao et al. [35] found that the possible reasons for the increase in sugar content during apple fruit development were related to the synthesis of Tre6P and trehalose. In this study, we found that Trehalose and Tre6P were both significantly higher in mature PCA persimmons than in PCNA persimmons, that Tre6P may act in key signaling to regulate the metabolic pathways of sucrose and starch in persimmon fruit, and that trehalose can be degraded to produce large amounts of glucose under the catalysis of Treh.

RNA-seq data showed that the gene expressions encoding the starch synthesis enzymes (i.e., AGPase and GBE1) in PCNA and PCA persimmon mature fruits were significantly differentially expressed. From the summary of differential genes, it can be concluded that the conversion from sucrose to glucose may be the main difference in sugar metabolism between PCNA and PCA mature fruits by means of the trehalose pathway, cellulose degradation, β-D-glucosidase conversion, and glucose hydrolysis. β-glucosidase is a hydrolytic enzyme and is the rate-limiting enzyme in the cellulose degradation process [36]. During the metabolism of starch and sucrose in dragon fruit pulp, β-glucosidase mainly catalyzes the generation of cellobiose from fibrous dextrin, and cellobiose generates glucose under the catalysis of β-glucosidase [37]; in addition, β-glucosidase also catalyzes the generation of glucose from β-D-glucoside [38]. The number of downregulated genes encoding bglX (10 DEGs) was obviously higher than the number of upregulated genes (3 DEGs) between PCNA and PCA persimmons; In addition, glucan hydrolysis may play a role in the difference between PCNA and PCA persimmons. The eglC gene encodes an endoglucanase, and the expressions of the eglC gene were also upregulated in PCNA persimmons; starch degraded to dextrin, and then dextrin was converted to maltose and glucose [39]. The expression of GYG1, GBE1, ISA, and malQ se in PCNA persimmons was obviously downregulated (Figure 5b). Taken together, gene expressions that related to sucrose and starch metabolism (sucrose hydrolyses) in PCNA persimmon were significantly upregulated compared to PCA persimmons, and the results confirmed the downregulation of sucrose content. Interestingly, we found that PCNA persimmon glucose content was not higher than that of PCA under sucrose hydrolysis, probably because sucrose content is very low even if hydrolysis is not the main pathway of glucose synthesis, but this still needs further investigation. In conclusion, we suggest that sucrose catabolism in the starch and sucrose metabolic pathways may be the main difference in sugar metabolism between PCNA and PCA persimmons.

## 4. Materials and Methods

### 4.1. Plant Material

PCNA (varieties ‘Jiro’ and ‘Youhou’) and PCA (variety ‘Zhongshi No.5’) persimmons were planted in the forest planting base managed by the Research Institute of Non-timber Forestry (34°55′18″–34°56′27″ N, 113°46′14″–113°47′35″ E), Yuanyang County, Henan Province, China. These 10-year-old cultivars were managed using conventional cultivation measures, with a row spacing of 3 × 4 m. Persimmon fruit without astringency were collected during the matured-fruit phase. To ensure sample consistency, completely matured PCA persimmons on the trees were collected when they lost enough astringency to be eaten. The fruits of three varieties were randomly collected from the three clones with each replicate consisting of ten fruits. Until they could be processed for metabolic detection and RNA extraction, the flesh in the equatorial plane was flash-frozen within liquid nitrogen and kept at −80 °C in a refrigerator.

### 4.2. Soluble Sugar, Starch, and Sucrose Synthase and Invertase Activity Measurement

The soluble sugar content in persimmon fruit was measured according to previous studies with some modifications [40]. Approximately 1 g of fruit was grounded into powder and then extracted in 80 °C water using 4.5 mL of Milli-Q^®^ ultrapure water for 30 min. After being chilled, the sample was centrifuged at 10,000 rpm for 20 min. The pellet extraction was performed once again, and the supernatant was saved. After combining the supernatants, the final volume was 10 mL. High-performance liquid chromatography (HPLC) with a CNW Athena NH2-RP column (4.6 × 250 mm, 5 µm) was used to determine the concentration of glucose and fructose together with the following parameters: injection volume = 10 µL, mobile phase = 75% acetonitrile, flow rate = 1.0 µL/min, and column heater temperature = 40 °C. An external reference fructose and glucose solution acquired from Beijing Solarbio Science & Technology Co., Ltd. (Beijing, China) was employed for determining the total amount of sugar in all samples. By comparing each sample’s peak area and retention duration to that of a calibrated sugar solution, we could determine each sample’s concentration. Each experiment had four independent replications.

Starch content was estimated using anthrone colorimetry by Cao et al. [41]. A fruit sample of approximately 1.0 g was placed in a prechilled mortar, ground into a homogenate with 80% (*v*/*v*) ethanol on an ice bath and transferred into a stoppered test tube. Then, 9 mL of 80% (*v*/*v*) ethanol was added, and the mixture was boiled in a boiling water bath for 30 min. Afterwards, it was removed and cooled in ice water, then it was centrifuged at a low temperature of 8000 r/min for 20 min, and the supernatant was discarded. An amount of 9 mL of 80% (*v*/*v*) ethanol was added again and the above steps were repeated once. The residue was dried, then 2 mL of distilled water was added. It was boiled in a boiling water bath for 15 min and cooled in ice-cold water. Then, 2 mL of cold 9.2 mol/L perchloric acid (HClO_4_) was added for 15 min, followed by an addition of 6 mL of distilled water, mixing and centrifuging at a low temperature of 8000 r/min for 10 min, and the transference of the supernatant to a 25 mL volumetric flask. An amount of 2 mL of cold 4.6 mol/L HClO_4_ was added to the filter residue for 15 min, then 6 mL of distilled water was added, followed by centrifugation for 20 min and the transference of the supernatant to a 25 mL volumetric flask. The precipitate was washed twice with 2 mL of distilled water, followed by centrifugation and the transference of the supernatant to the volumetric flask. Finally, the volumetric flask was filled with distilled water to 25 mL, resulting in the starch extraction solution. The detection of starch was performed at 620 nm with a visible ultraviolet spectrophotometer UV. Starch content was determined according to an external sucrose standard solution (Beijing Solarbio Science & Technology Co., Ltd.).

The enzyme activities of NI, AI, sucrose synthase-invertase (SS-I), SS, and SPS were determined by using the kit purchased from Beijing Solarbio Science & Technology Co., Ltd. (BC0565, BC0575, BC0585, BC0605, and BC4315). These assays were conducted according to the protocol of the correlated kit.

### 4.3. Transcriptome Sequencing

Total RNA was isolated with TRIzol Reagent (B511321; Sangon, Shanghai, China). Paired-end sequencing libraries were prepared with three biological replicates for each sample and subsequently placed for sequencing through the Illumina™ NovaSeq^®^ platform (Illumina™, San Diego, CA, USA). We used the hexaploid persimmon genome (*D. kaki* (variety ‘Xiaoguo-tianshi’, unpublished) as a reference sequence for alignment and subsequent analysis. Sequencing reads and read alignments were compared using HISAT2 [42] and assembled using StringTie. To determine how many reads were mapped to each gene, we utilized FeatureCounts^®^ v1.5.0-p3 to further predict new transcripts [43]. The Fragments Per Kilobase of Transcript per Million Mapping Reads (FPKM) was then calculated by multiplying its length by the number of mapped reads. We used DESeq2 (1.18.0) to compare the expression levels of two distinct groups [44]. Genes that had a padj ≤0.05 and |log2-fold change| ≥ 1 were considered DEGs.

### 4.4. Metabolite Profiling Analysis

Extracts of 100 mg of persimmon fruit powder were made by vortexing 500 μL of 80% (*v*/*v*) prechilled methanol. There were 3 biological replicates for each sample. Then, 500 μL of the supernatant from centrifugation (15,000× *g*, 4 °C/20 min) was diluted into 53% methanol using Milli-Q^®^ ultrapure water. After that, the samples were filtered (0.22 μm membrane filter) and subjected to centrifuging (15,000× *g* for 20 min at 4 °C). The persimmon fruit extract samples were separated through the ExionLCTM AD system (SCIEX™) connected to a QTRAP^®^6500+ mass spectrometer (SCIEX™) and fitted with an Xselect HSS T3 column (2.1 × 150 mm, 2.5 μm). The column temperature was 50 °C, the injection volume was 1.5 μL, and the flow rate was 0.4 mL/min; these were the settings used for the analysis. Water was the mobile phase. The gradient program for phase A/phase B was 98:2 (*v*/*v*) at 0 min, 98:2 (*v*/*v*) at 2 min, 0:100 (*v*/*v*) at 15 min, 0:100 (*v*/*v*) at 17 min, 98:2 (*v*/*v*) at 17.1 min, and 98:2 (*v*/*v*) at 20 min.

To check the system’s consistency and the experimental data’s accuracy, samples were placed into quality control (QC) within a queue mode. Electrospray ionization (ESI) source settings allowed each sample to be run in both negative and positive ion modes. Mass spectrum databases from the MRM (Multiple Reaction Monitoring) of the Novogene in-house database were consulted to compare the spectra and the retention index (RI) with the reference compounds previously examined with the same system. KEGG (http://www.genome.jp/kegg/) [45], HMDB (http://www.hmdb.ca/) [46], and Lipidmaps databases (http://www.lipidmaps.org/) [47] were used for metabolite annotation. Principal components analysis (PCA) was performed at metaX [48]. Differential metabolites were defined as those with a *p*-value < 0.05 and fold change ≥ 2.

### 4.5. Quantitative RT-PCR Assessments

The cDNA used in the RNA-seq study was converted from total RNA through a TRUE-script First-Strand^®^ cDNA Synthesis Kit (Kemix™, Beijing, China). RT-qPCR runs were conducted with the LightCycler 480 II (Roche), using 96-well plates. Each gene underwent a three-minute reaction at 95 °C, with 45 subsequent cycles of 5 s at that temperature and 30 cycles at 55–60 °C. Using glyceraldehyde-3-phosphate dehydrogenase (GAPDH) as a reference gene [49], the 2^−∆∆Ct^ technique determined the relative expression for each gene, and three replicates of each reaction were performed. A Pearson’s correlation assessment was conducted through SPSS^®^ (v 24.0; SPSS Inc.™, Chicago, IL, USA). The RT-qPCR primers are listed in Table S1.

### 4.6. Statistical Analysis

ANOVA and the post hoc test were performed using Excel 2019 and SPSS 24.0 software. The Pearson correlation analysis was carried out through Hiplot [50].

## 5. Conclusions

In conclusion, a comprehensive transcriptomic and metabolomic analysis of PCNA (‘Jiro’ and ‘Yohou’) and PCA (‘Zhongshi No.5′) persimmon fruit was conducted. The concentration of soluble sugar, starch content, and sucrose synthase and invertase of PCNA and PCA persimmons were significantly different. For example, the fructose, glucose, SS-I, and AI activity levels in the PCNA persimmon fruit were markedly lower than those in the PCA persimmon fruit. Through the KEGG enrichment analysis, it was found that the sucrose and starch metabolic pathways were highly enriched in the PCNA and PCA fruit. For the starch and sucrose metabolism pathway, 11 metabolites were identified. Moreover, most of these differentially accumulated sucrose metabolites were related to trehalose synthesis, indicating that the trehalose synthesis pathway might influence the sugar variation between PCNA and PCA persimmons. In addition, the expression patterns of deferentially expressed genes (such as *bglX*, *eglC*, *Cel*, *TPS*, *SUS*, and *TREH* genes) were significantly correlated with the content of deferentially accumulated metabolites (such as starch, sucrose, and trehalose). These results showed that the sucrose and starch metabolism pathway maintained a central position of sugar variation between PCNA and PCA fruit. This study provides basic information and useful resources for future studies on the influence of astringency types on sugar differences between PCNA and PCA persimmon fruit.

## Data Availability

The transcriptome sequencing raw data were deposited in the National Center for Biotechnology Information Sequence Read Archive (NCBI SRA) under the Bioproject ID PRJNA897083.

## Supplementary Materials

The supporting information can be downloaded at: https://www.mdpi.com/article/10.3390/ijms24108599/s1.
