# Supplementary figures and images for: Transcriptome and Metabolome Reveal Distinct Sugar Accumulation Pattern between PCNA and PCA Mature Persimmon Fruit
Source: Int J Mol Sci. 2023 May 11;24(10):8599. doi: 10.3390/ijms24108599 (PMC10217969; doi:10.3390/ijms24108599)

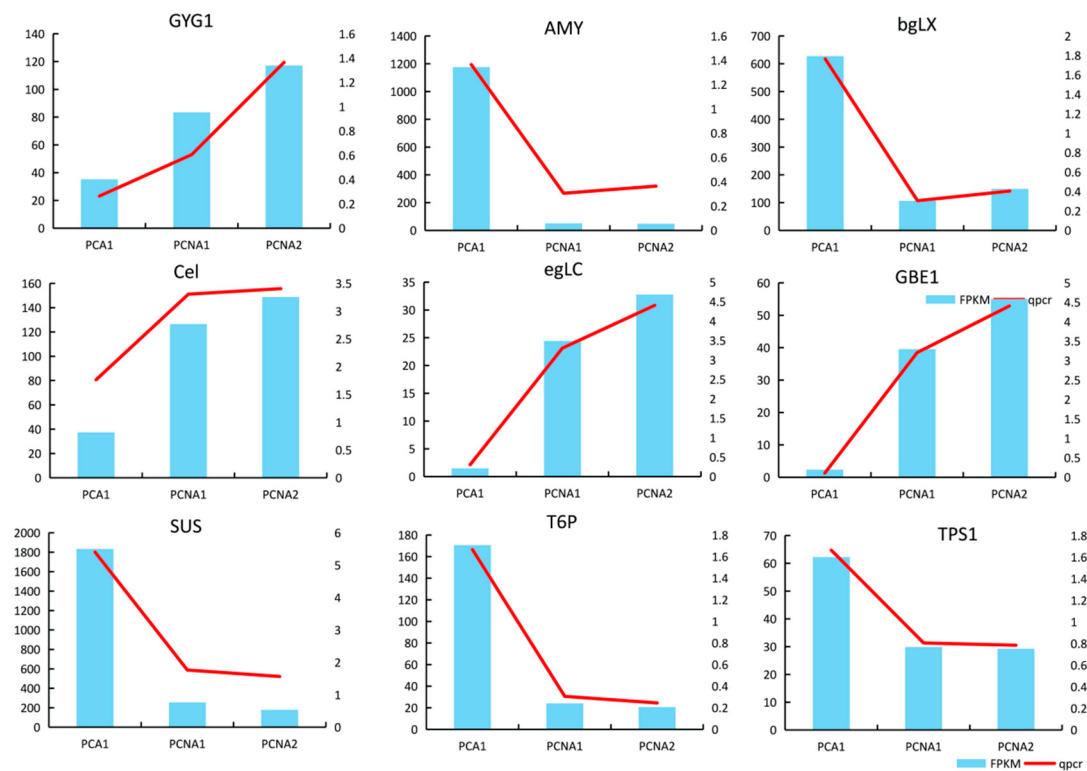

Figure S1. Verification of transcriptomic data by qRT-PCR analysis of 9 gene expression

Supplement: Supplementary file 1 [file ijms-24-08599-s001.zip › supplmentary figure.pdf]
